# Supplementary material for: A Global Screen for Assembly State Changes of the Mitotic Proteome by SEC-SWATH-MS
Source: Cell Syst. 2020 Feb 26;10(2):133–155.e6. doi: 10.1016/j.cels.2020.01.001 (PMC7042714; doi:10.1016/j.cels.2020.01.001)

Q9P2K8 | E2AK4_HUMAN | EIF2AK4 GCN2 KIAA1338
Monomer MW [kDa]: 186.911 Monomer expected elution fraction: 35

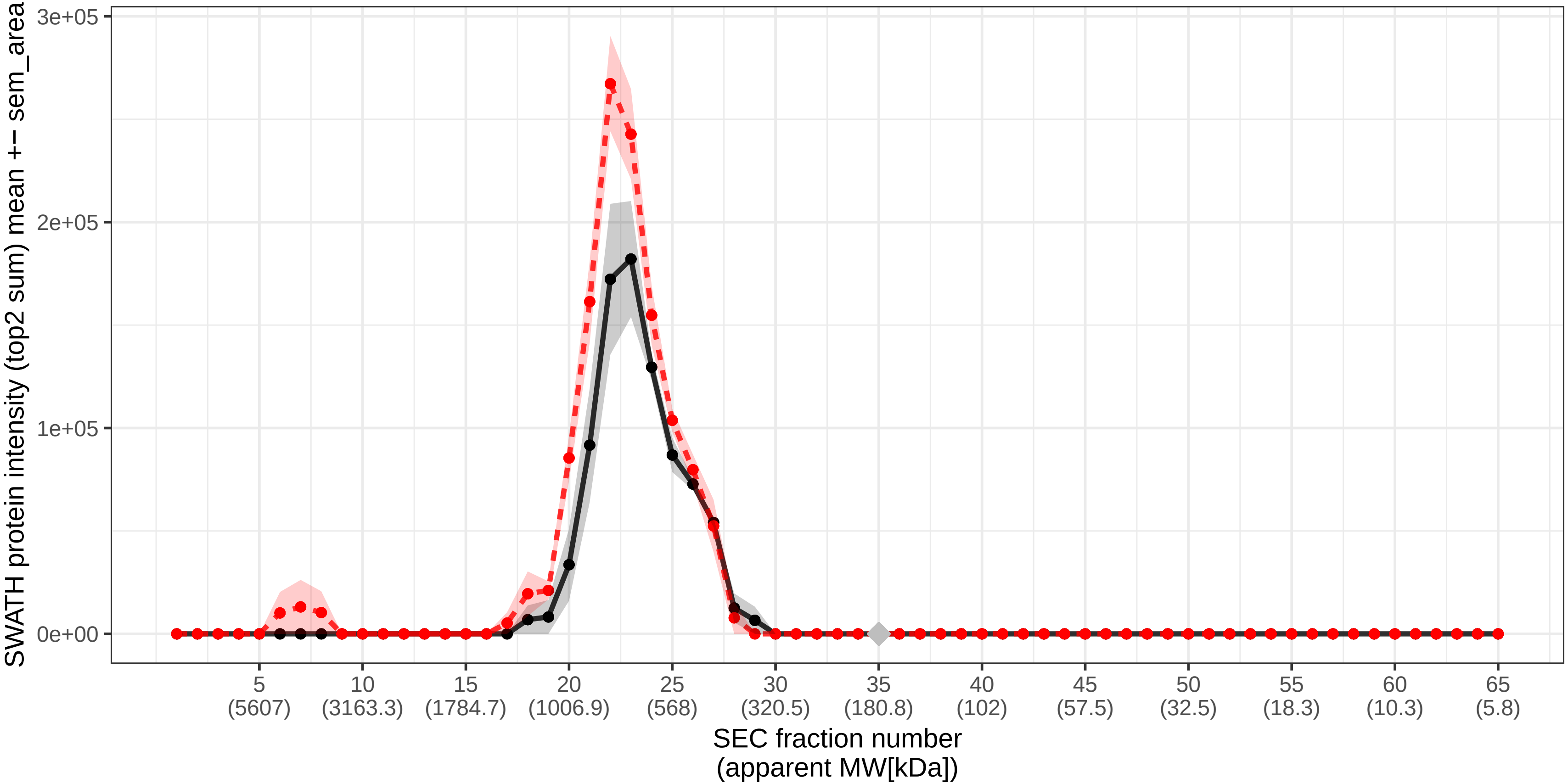

Supplement: Data S1. SEC-SWATH-MS Protein Chromatograms, Related to Figure 1 [file mmc6.zip › SECchrom_Q9P2K8_E2AK4_HUMAN_EIF2AK4_GCN2_KIAA1338.pdf]
